# Unhappiness with the Fetal Gender is associated with Depression in Adult Pregnant Women Attending Prenatal Care in a Public Hospital in Durango, Mexico

**Published:** 2016-03

**Authors:** Cosme Alvarado-Esquivel, Antonio Sifuentes-Alvarez, Carlos Salas-Martinez

**Affiliations:** 1 Biomedical Research Laboratory, Faculty of Medicine and Nutrition, Juárez University of Durango State. Durango, Mexico;; 2 General Hospital, Secretary of Health, Durango. Mexico

**Keywords:** Depression, Pregnancy, adults, cross-sectional, epidemiology, Mexico

## Abstract

Depression during pregnancy has been scantily studied in Mexican women. We aimed to determine the prevalence and correlates of depression in adult pregnant women attending a public hospital in the northern Mexican city of Durango, Mexico. Through a cross-sectional study design, we assessed depression in 270 adult pregnant women attended for prenatal care in a public hospital using a validated Mexican version of the Edinburg Postnatal Depression Scale in pregnancy and further confirmation by a psychiatric evaluation using the DSM-IV criteria for depression. Prevalence association with socio-demographic, clinical and psychosocial characteristics of the pregnant women was also investigated. Of the 270 pregnant women studied, 101 (37.4%) had EPDS scores equal to or higher than nine. Depression was confirmed in 56 (20.7%) women. Of them, 42 suffered from minor depression and 14 from major depression. Multivariate analysis of socio-demographic, clinical and psychosocial characteristics of the women showed that depression was associated with depression before pregnancy (OR = 3.36; 95% CI: 1.20-9.40; *P*=0.02), anxiety during pregnancy (OR = 9.38; 95% CI: 1.87-46.96; *P*=0.006), smoking (OR = 25.05; 95% CI: 1.77-353.07; *P*=0.01), unhappy with the fetal sex (OR = 8.53; 95% CI: 2.46-29.48; *P*<0.001), and unintended pregnancy (OR = 2.90; 95% CI: 1.07-7.86; *P*=0.03). Results indicate that about one fifth of the pregnant women studied had confirmed depression. This is the first report of an association of prenatal depression with unhappiness with the fetal sex. Factors associated with prenatal depression found in this study may help for the optimal design of preventive measures against prenatal depression.

## INTRODUCTION

Depression during pregnancy is highly prevalent and understudied (1). Depression may occur in about 20% of pregnant women (2). However, only a small number of women with major depression seek treatment during pregnancy and postpartum (3). Depression during pregnancy may lead to morbidity in both mothers and offspring (1, 4). Depression during pregnancy has been associated with obstetric complications (5), premature delivery and decreased breastfeeding initiation (6). The presence of depression during pregnancy is a strong predictor of postnatal depression (7, 8) and it is more common than postpartum depression (8). Prenatal depression may also lead to postpartum psychosis (9). A number of risk factors have been associated with prenatal depression including life stress, lack of social support, domestic violence (10), poverty, unplanned pregnancy and marital difficulties (2). The Edinburgh Postpartum Depression Scale (EPDS) has been used to screen for depression during pregnancy and postpartum (3).

Very little is known about the epidemiology of prenatal depression in Mexico. Prevalences of depression in pregnant women in Mexico City varied from 16.6% to 36.8% (11, 12). Whereas, in a previous study in the southern Mexican city of Cancun, Quintana Roo, researchers found a low (6.4%) prevalence of depression in pregnant women (13). We sought to determine the prevalence of depression in pregnant women attended for prenatal care in a public hospital in Durango City, Mexico; and to determine the correlates of prenatal depression in the women studied.

## METHODS

### Selection and description of participants

Through a cross-sectional study design, we studied adult pregnant women who attended routine prenatal consultations in a public hospital (General Hospital of the Secretary of Health) in Durango City, Mexico. Sampling was performed at random from March 2013 to October 2014. Inclusion criteria for enrollment in the study were pregnant women (2-9 month of pregnancy) attended for prenatal care in the General Hospital, aged 18 years and older, and who accepted to participate. Occupation, socioeconomic status or residence of the women were not restrictive criteria for enrollment. In total, 270 women were included in the study. They were 18-45 years old (mean age: 27.41 ± 6.66 years) and had a low socioeconomic status. Participants were evaluated within their 2-9 months (median: 8 months) of pregnancy. Of the 270 women studied, 52 were in their first pregnancy and 218 were in their 2-8 pregnancy.

### Technical information

We used a validated Mexican version of the EPDS (14) for screening depression in pregnant women. An EPDS cut-off of 9/10 was used to screen depression since this cut-off had the best performance in Mexican adult pregnant women (14). Participants who scored nine or higher in the EPDS were interviewed by a psychiatrist to examine major and minor depression by using the DSM-IV criteria (15). Depression in a woman was considered only when the psychiatrist confirmed the diagnosis. The psychiatrist who assessed depression in the pregnant women was blind to the EPDS scores. The psychiatric consultations were performed during the same day the EDPS were submitted.

### Socio-demographic, clinical and psychosocial characteristics of the pregnant women

We obtained the socio-demographic, clinical and psychosocial characteristics of the pregnant women with the aid of a questionnaire. Socio-demographic items included age, occupation, marital status, education, birthplace, residence, religion, having a health insurance, age at marriage, and number of marriages. Clinical data included health status, obstetric history, type of obstetric outcome (delivery or cesarean section) of last pregnancy, presence of complications during their last delivery, history of breastfeeding, health status of their last newborn, number of children, history of depression before pregnancy or trauma in live, history of postpartum depression, stress or anxiety before pregnancy, gestational age, number of fetuses in the current pregnancy, knowledge of the fetal sex, fetal sex, a previous depression episode during pregnancy, anxiety or stress during pregnancy, smoking, consumption of alcohol, drug abuse, and size and health status of the fetus. Psychosocial data included separation from parents at young age, presence of financial or family problems, bad relation with her mother in law, satisfaction with her education, support from her couple, relatives, friends or colleagues, support from the Mexican government, happiness for the sex of the fetus, intended pregnancy, good relation with her couple, living with her couple, abandoned by her couple, violence from her couple, satisfaction with her body image, and couple living abroad.

### Statistics

Results were analysed with the SPSS version 15.0 software. Bivariate analysis was used to select variables for multivariate analysis. The association between depression and the characteristics of the pregnant women was assessed by multivariate analyses. For initial comparison of frequencies among groups, we used the Pearson´s chi square and the Fisher exact test (when values were small). Variables with a *P* value equal to or less than 0.05 obtained in the bivariate analysis were included in the multivariate analysis. Odd ratios (OR) and 95% confidence intervals (CI) were calculated by multivariate analysis using the Enter method. The Hosmer-Lemeshow goodness of fit test was used to assess the fitness of our regression model. A *P* value less than 0.05 was considered as statistical significant.

### Ethical aspects

The purpose and procedures of the study were explained to all participants. All participants were adults and a written informed consent was obtained from all of them. The Ethical Committee of the General Hospital of the Secretary of Health in Durango City, Mexico approved this survey. This study was conducted in accordance with the principles of the Declaration of Helsinki.

## RESULTS

Of the 270 pregnant women studied, 101 (37.4%) had EPDS scores equal to or higher than 9 (range 9-25). These 101 women with likely presence of depression detected by the screening test were further examined for confirmation of depression. The psychiatric evaluation confirmed depression in 56 of the 101 women. Thus, the general prevalence of depression in the pregnant women studied was 20.7%. Of the 56 women with depression, 42 suffered from minor depression and 14 suffered from major depression. Therefore, the prevalence of minor and major depression in the women studied were 15.6% and 5.2%, respectively. Depressed women were treated with psychotherapy or sertraline.

Of the socio-demographic characteristics of women, the frequency of depression increased with the number of marriages (*P*=0.001). Other socio-demographic characteristics of women including age, occupation, marital status, educational level, birthplace, residence, religion, having a health insurance, or age at marriage showed *P* values higher than 0.05.

With respect to the clinical characteristics, bivariate analysis showed a number of variables with a *P* value equal to or lower than 0.05: history of cesarean section (*P*=0.01), depression before pregnancy (*P*<0.001), history of trauma (*P*=0.001), stress (*P*<0.001) and anxiety (*P*<0.001) before pregnancy, a previous depression episode during pregnancy (*P*=0.001), anxiety (*P*=0.001) and stress (*P*=0.001) during pregnancy, smoking (*P*=0.003), alcohol consumption (*P*=0.03), and fetal size (*P*=0.04). Other clinical characteristics of women including health status, number of pregnancies, deliveries, miscarriages or stillbirths, outcome of last pregnancy, presence of complications during their last delivery, history of breastfeeding, health status de their last newborn, number of children, history of postpartum depression, trimester of pregnancy, number of fetuses in the current pregnancy, knowledge of fetal sex, fetal sex, health status of the fetus, or drug abuse showed *P* values >0.05 by bivariate analysis.

Concerning psychosocial characteristics of the women, bivariate analysis showed some variables with *P* values equal to or lower than 0.05: presence of financial (*P*=0.001) and family (*P*=0.002) problems, unhappiness for the gender of the fetus (*P*=0.002), unintended pregnancy (*P*=0.006), bad relation with her couple (*P*=0.02), violence from her couple (*P*=0.02), and unsatisfied with her body image (*P*=0.004). Other psychosocial characteristics of the women including separation from parents at young age, bad relation with her mother in law, satisfaction with her education, support from her couple, relatives, friends or colleagues, support from the Mexican government, living with her couple, ever abandoned by her couple, and couple living abroad showed *P* values >0.05 by bivariate analysis. Multivariate analysis (Table 1) of socio-demographic, clinical and psychosocial characteristics of the women with *P* values equal to or lower than 0.05 by bivariate analysis showed that depression was associated with depression before pregnancy (OR = 3.36; 95% CI: 1.20-9.40; *P*=0.02), anxiety during pregnancy (OR = 9.38; 95% CI: 1.87-46.96; *P*=0.006), smoking (OR = 25.05; 95% CI: 1.77-353.07; *P*=0.01), unhappiness with the fetal sex (OR = 8.53; 95% CI: 2.46-29.48; *P*<0.001), and unintended pregnancy (OR = 2.90; 95% CI: 1.07-7.86; *P*=0.03). The result of the Hosmer-Lemeshow test (*P*=0.39) indicated an acceptable fit of our regression model.

**Table 1:** Multivariate analysis of selected characteristics of pregnant women and their association with depression

Characteristic	Odds ratio	95% confidence interval	*P* value

Number of marriages	1.23	0.51-2.95	0.63
History of cesarean sections	1.42	0.84-2.41	0.18
Depression before pregnancy	3.36	1.20-9.40	0.02
Trauma in life	0.94	0.31-2.82	0.92
Stress before pregnancy	0.67	0.17-2.61	0.56
Anxiety before pregnancy	1.98	0.49-7.95	0.33
Previous depression during pregnancy	0.80	0.21-2.95	0.73
Anxiety during pregnancy	9.38	1.87-46.96	0.006
Stress during pregnancy	1.13	0.27-4.75	0.85
Smoking	25.05	1.77-353.07	0.01
Alcohol consumption	0.24	0.02-2.53	0.24
Small fetal size	1.24	0.19-7.88	0.81
Financial problems	2.18	0.82-5.79	0.11
Family problems	1.60	0.45-5.67	0.45
Unhappy with the fetal sex	8.53	2.46-29.48	<0.001
Unintended pregnancy	2.90	1.07-7.86	0.03
Bad relation with her couple	0.39	0.11-1.38	0.14
Violence from her couple	0.99	0.31-3.12	0.99
Unsatisfied with her body image	0.38	0.11-1.25	0.11

## DISCUSSION

Very little is known about depression in pregnant women in Mexico. In the present study, we determined the prevalence and correlates of depression in adult pregnant women attending a public hospital in the northern Mexican city of Durango. Results showed that 20.7% of the pregnant women studied suffered from depression. This prevalence is higher than the 6.4% prevalence of depression in pregnant women in the southern Mexican city of Cancun, Quintana Roo (13). It is not clear why the pregnant women of Durango had a higher prevalence of depression than that in women in Cancun. However, it is likely that difference in socio-demographic characteristics among the populations and difference in methods among the studies may explain the difference in the prevalences. We included only adult women whereas researchers in Cancun included both adult and adolescent women. We did not exclude women with history of depression whereas the other research group excluded women who have suffered from depression 6 months before pregnancy. On the other hand, the prevalence of depression found in our study is lower than the 32.5% prevalence of depression reported in pregnant women in Mexico City (16). However, this comparison should be interpreted with care since pregnant women studied in Mexico City were adolescents while we studied adult pregnant women. In a further study of prenatal depression in pregnant Latinas in the USA and Mexico City, researchers found depressive symptoms in 32.4% of Latinas and in 36.8% of Mexicans by using the Center for Epidemiological Studies Depression Scale (11). In a recent study, 16.6% of 210 pregnant women in Mexico City had depressive symptoms by using the Patient Health Questionnaire (PHQ-9) (12). However, the use of different depression scales among the studies makes the prevalence comparison unfair. We studied women in Durango because they have a number of characteristics that are different from those of women living in other Mexican states. For instance, poverty in women in Durango is common (Durango State is the poorest state in northern Mexico); migration in Durango is also common and many women live without their partners; and violence in Durango is also high. All these characteristics might contribute for depression in women. There are numerous tools to screen prenatal depression (3). However, to the best of our knowledge this is the first study to determine the prevalence and correlates of prenatal depression in Mexico by using the EPDS. In an international context in studies using the EPDS, the prevalence found in the present study is similar than the 19.9% prevalence reported in pregnant women in Ethiopia (13). In contrast, the prevalence found in our study is lower than the 30% prevalence found in Hispanic women in Western Massachusetts, USA (18). Prevalence result of the present study is in line with others in socially vulnerable women.

We searched for characteristic of pregnant women associated with depression. Multivariate analysis of socio-demographic, clinical and psychosocial characteristics of the women showed that depression was associated with depression before pregnancy, anxiety during pregnancy, smoking, unhappiness with the fetal sex, and unintended pregnancy. In a meta-analysis of 57 studies to evaluate risk factors for antepartum depressive symptoms, the variable history of depression was associated with prenatal depression by bivariate analysis (10). In the present study, the variable depression before pregnancy was found associated with prenatal depression in both bivariate and multivariate analyses. Prenatal depression has been linked with anxiety. In a study of pregnant women in a socioeconomically deprived area in Adelaide, Australia, researchers found an increased risk of antenatal depression with previous feelings of depression and anxiety (19). In addition, anxiety during pregnancy has been linked to intense postnatal depressive symptoms (20). The association of depression with smoking found in our study is consistent with previous observations. In a Brazilian study of pregnant women, the presence of depression was associated with smoking during pregnancy (21). Similarly, smoking was a significant, independent predictor of prenatal depression symptoms in a study of pregnant women in New York, USA (22). Interestingly, exposure to environmental tobacco smoke has been linked with increased risk for depressive symptoms during pregnancy too (23). In a recent study in the USA, the suggestion of an association of failure to discontinue cigarette smoking at the onset of pregnancy with higher risk of prenatal depressive symptoms among Hispanic women was made (18). The finding of an association of depression with the variable “unhappiness with the fetal sex” was expected since an association of the sex of the infant with postnatal depression has been reported (24). However, to the best of our knowledge, this is the first time an association of depression during pregnancy with the factor “unhappiness with the fetal sex” is reported. The association of prenatal depression with unintended pregnancy found in the present study is consistent with results of other studies. Unplanned pregnancy has been identified as an important variable determining higher severity of anxiety and depression during pregnancy (25). In a study in New York, USA, researchers found that women with unintended pregnancies were more likely to report severe or moderate prenatal depression symptoms compared to women with intended pregnancies (22).

## CONCLUSIONS

Results indicate that about one fifth of the adult pregnant women studied had confirmed depression. This is the first report of an association of prenatal depression with unhappiness with the fetal sex. Further research to confirm this association is needed. Factors associated with prenatal depression found in the present study may help for the optimal design of preventive measures against depression in pregnant women.

## ABBREVIATIONS

EPDS: Edinburg Postpartum Depression Scale
OR: Odds ratio
CI: Confidence interval
SPSS: Statistical Package for the Social Sciences
USA: United States of America

## References

[1] Davalos DB, Yadon CA, Tregellas HC (2012). Untreated prenatal maternal depression and the potential risks to offspring: a review. Arch. Womens Ment. Health.

[2] Bowen A, Muhajarine N (2006). Antenatal depression. Can. Nurse.

[3] Marcus SM (2009). Depression during pregnancy: rates, risks and consequences--Motherisk Update 2008. Can. J. Clin. Pharmacol.

[4] Waters CS, Hay DF, Simmonds JR, van Goozen SH (2014). Antenatal depression and children's developmental outcomes: potential mechanisms and treatment options. Eur. Child Adolesc. Psychiatry.

[5] Alder J, Fink N, Bitzer J (2007). Depression and anxiety during pregnancy: a risk factor for obstetric, fetal and neonatal outcome? A critical review of the literature. J. Matern. Fetal Neonatal Med.

[6] Grigoriadis S, VonderPorten EH, Mamisashvili L (2013). The impact of maternal depression during pregnancy on perinatal outcomes: a systematic review and meta-analysis. J. Clin. Psychiatry.

[7] Alvarado-Esquivel C, Sifuentes-Alvarez A, Estrada-Martínez S (2010). Prevalence of postnatal depression in women attending public hospitals in Durango, Mexico. Gac. Med. Mex.

[8] Field T (2011). Prenatal depression effects on early development: a review. Infant Behav. Dev.

[9] Ebeid E, Nassif N, Sinha P (2010). Prenatal depression leading to postpartum psychosis. J. Obstet. Gynaecol.

[10] Lancaster CA, Gold KJ, Flynn HA (2010). Risk factors for depressive symptoms during pregnancy: a systematic review. Am. J. Obstet. Gynecol.

[11] Lara MA, Le HN, Letechipia G, Hochhausen L (2009). Prenatal depression in Latinas in the U.S. and Mexico. Matern. Child Health J.

[12] Lara MA, Navarrete L, Nieto L (2015). Prevalence and incidence of perinatal depression and depressive symptoms among Mexican women. J. Affect Disord.

[13] Ceballos-Martínez I, Sandoval-Jurado L, Jaimes-Mundo E (2010). Epidemiological characteristics of depressed Mexican pregnant women. Rev. Med. Inst. Mex. Seguro. Soc.

[14] Alvarado-Esquivel C, Sifuentes-Alvarez A, Salas-Martinez C (2014). Validation of the Edinburgh postpartum depression scale in a population of adult pregnant women in Mexico. J. Clin. Med. Res.

[15] Pichot P, López-ibor Aliño JJ, Valdés Miyar M (1995). In DSM-IV. Manual diagnóstico y estadístico de los trastornos mentales. Barcelona. Masson, SA.

[16] Lara MA, Berenzon S, Juárez García F (2012). Population study of depressive symptoms and risk factors in pregnant and parenting Mexican adolescents. Rev. Panam. Salud. Publica.

[17] Dibaba Y, Fantahun M, Hindin MJ (2013). The association of unwanted pregnancy and social support with depressive symptoms in pregnancy: evidence from rural Southwestern Ethiopia. BMC Pregnancy Childbirth.

[18] Fortner RT, Pekow P, Dole N (2011). Risk factors for prenatal depressive symptoms among Hispanic women. Matern Child Health J.

[19] Edwards B, Galletly C, Semmler-Booth T, Dekker G (2008). Antenatal psychosocial risk factors and depression among women living in socioeconomically disadvantaged suburbs in Adelaide, South Australia. Aust. N Z J. Psychiatry.

[20] Sutter-Dallay AL, Giaconne-Marcesche V, Glatigny-Dallay E, Verdoux H (2004). omen with anxiety disorders during pregnancy are at increased risk of intense postnatal depressive symptoms: a prospective survey of the MATQUID cohort. Eur. Psychiatry.

[21] Silva RA, Jansen K, Souza LD (2010). Depression during pregnancy in the Brazilian public health care system. Rev. Bras. Psiquiatr.

[22] Fellenzer JL, Cibula DA (2014). Intendedness of pregnancy and other predictive factors for symptoms of prenatal depression in a population-based study. Matern Child Health J.

[23] Mbah AK, Salihu HM, Dagne G (2013). Exposure to environmental tobacco smoke and risk of antenatal depression: application of latent variable modeling. Arch. Womens Ment Health.

[24] Goldbort J (2006). Transcultural analysis of postpartum depression. MCN Am. J. Matern Child Nurs.

[25] Morylowska-Topolska J, Makara-Studziñska M, Kotarski J (2014). The influence of sociodemografic and medical variables on severity of anxiety and depressive symptoms during particular trimesters of pregnancy. Psychiatr Pol.

